# Performance of prognostic models in critically ill cancer patients – a review

**DOI:** 10.1186/cc3765

**Published:** 2005-07-08

**Authors:** Sylvia den Boer, Nicolette F de Keizer, Evert de Jonge

**Affiliations:** 1 Intensivist, Department of Intensive Care, Academic Medical Center, Universiteit van Amsterdam, Amsterdam, Netherlands; 2 Informatician, Department of Medical Informatics, Academic Medical Center, Universiteit van Amsterdam, Amsterdam, Netherlands

## Abstract

**Introduction:**

Prognostic models, such as the Acute Physiology and Chronic Health Evaluation (APACHE) II or III, the Simplified Acute Physiology Score (SAPS) II, and the Mortality Probability Models (MPM) II were developed to quantify the severity of illness and the likelihood of hospital survival for a general intensive care unit (ICU) population. Little is known about the performance of these models in specific populations, such as patients with cancer. Recently, specific prognostic models have been developed to predict mortality for cancer patients who are admitted to the ICU. The present analysis reviews the performance of general prognostic models and specific models for cancer patients to predict in-hospital mortality after ICU admission.

**Methods:**

Studies were identified by searching the Medline databases from 1994 to 2004. We included studies evaluating the performance of mortality prediction models in critically ill cancer patients.

**Results:**

Ten studies were identified that evaluated prognostic models in cancer patients. Discrimination between survivors and non-survivors was fair to good, but calibration was insufficient in most studies. General prognostic models uniformly underestimate the likelihood of hospital mortality in oncological patients. Two versions of a specific oncological scoring systems (Intensive Care Mortality Model (ICMM)) were evaluated in five studies and showed better discrimination and calibration than the general prognostic models.

**Conclusion:**

General prognostic models generally underestimate the risk of mortality in critically ill cancer patients. Both general prognostic models and specific oncology models may reliably identify subgroups of patients with a very high risk of mortality.

## Introduction

Advances in oncological and supportive care have led to improved prognosis and extension of survival time in cancer patients. However, such advances have often been achieved through aggressive therapies and support, at high expense. Some of these patients require admission to the intensive care unit (ICU) for acute concurrent illness, postoperative care, or complications of their cancer or its therapy. Recent studies [1-6] suggest that mortality of cancer patients in the ICU is comparable with that of patient groups suffering from other severe diseases, but others reported a poor prognosis with much higher mortality rates [7]. Efforts have been made to identify parameters that are associated with poor prognosis and to develop scoring models for predicting hospital mortality at ICU admission of cancer patients.

Different prognostic systems, such as the Acute Physiology and Chronic Health Evaluation (APACHE) II or III [8,9], the Simplified Acute Physiology Score (SAPS) II [10], and the Mortality Probability Models (MPM) II [11], have been developed to predict the outcome of critically ill patients admitted to the ICU. Although these models perform well in predicting the mortality of the general ICU patient population, they may well under- or overestimate mortality in selected patient subpopulations that were not well represented in the original cohort on which the model was developed. Therefore, new models were designed for specific populations, such as cancer patients [12,13]. By including variables specific to oncology such as disease progression/recurrence, performance status and type of treatment, they were expected to perform better than the general models. The aim of this review is to evaluate the performance of the general severity-of-illness scores (APACHE II and III, SAPS II, MPM II) and the specific oncological scoring systems in cancer patients requiring admission to the ICU.

## Methods and materials

### Sources and selection criteria

The information in this review is based on results of a Medline search for recent studies published between 1994 and 2004. The key words used included "Severity of illness scores", "Acute Physiology and Chronic Health Evaluation (APACHE)", "Simplified Acute Physiology Score (SAPS)", "Mortality Probability Model II", "Cancer", "Oncology", "Critical care", "Prognosis and outcome" and "Hospital mortality". Based on the title and abstract of the publication, we selected English-language articles containing information on the performance of prognostic models in cancer patients admitted to ICUs. The references of all selected reports were cross-checked for other potentially relevant articles. It was envisaged that the studies would be too heterogeneous to combine for a formal meta-analysis and therefore a narrative synthesis was undertaken.

## Results

### Performance of the prognostic models

Although several measures exist for evaluating the performance of prognostic models, all identified studies used receiver operating characteristic (ROC) curves and the area under the curve (AUC) [14] to evaluate discrimination and the Hosmer-Lemeshow goodness-of-fit H- or Ĉ-statistics [15] to evaluate the calibration of the prognostic models.

'Discrimination' refers to a model's ability to distinguish survivors from non-survivors. The AUC represents the probability that a patient who died had a higher predicted probability of dying than a patient who survived. An AUC of 0.5 indicates that the model does not predict better than chance. The discrimination of a prognostic model is considered perfect if AUC = 1, good if AUC >0.8, moderate if AUC is 0.6 to 0.8, and poor if AUC <0.6 [16]. The AUC of a model gives no indication of how close the predicted probabilities are to the observed outcome. To take this aspect of a model's performance into account, we have to look at the calibration and accuracy of the prognostic models.

'Calibration' refers to the agreement between predicted probabilities and the 'true probabilities'. Of course, the true probability of a patient's outcome is not known, otherwise there would be no need to develop prognostic models. However, the true probabilities can be approximated by taking the mean of the observed outcomes within predefined groups of patients. The selected studies used Hosmer-Lemeshow H- or Ĉ-statistics. Both H- and Ĉ-statistics compare the observed mortality in a group with the predicted mortality of that group. A disadvantage of the Hosmer-Lemeshow tests is that the value of the statistic is sensitive to the choice of the cut-off points that define the groups. The H- and Ĉ-statistics differ in the way the groups of patients are composed [15]. Grouping for the H-statistic is based on partitioning of the probability interval (0–1) into ten equally sized ranges. The Ĉ goodness-of-fit statistic sorts observations according to their expected probability and partitions the observations into ten groups of equal size. A high H or Ĉ relates to a small *p *value, implying significant difference between observed and predicted mortality, and thus indicates a lack of fit of the model. It is a generally known weakness of the Hosmer-Lemeshow goodness-of-fit statistics that the sample size has a major influence on the measured calibration. Using small samples will result in an apparently good fit, using large samples will result in an apparently poor fit [17,18].

Discrimination and calibration describe the overall predictive power of a model. This is important when analyzing the mortality risk of a population, for example, to determine performance of an ICU by measuring the standardized mortality ratio (SMR: observed mortality divided by predicted mortality) as mortality adjusted for severity-of-illness. When caring for an patient, it is more important that a model can reliably predict the likelihood of an outcome of an individual patient; this is called 'accuracy'. Accuracy refers to the difference between predictions and observed outcomes at the level of individuals. The mean squared error (MSE), also called Brier score, is an example of an accuracy measure [19]. None of the selected studies evaluated accuracy measures. However, some studies notify that when caring for an individual patient, it is more important that a model can reliably identify patients with a very high risk of dying. Therefore, they evaluated the performance of prognostic models at specific cut-off points, dividing high-risk patients from low-risk patients.

### Discrimination

Nine studies reported on discriminating ability of general prognostic models in ICU patients with cancer (Table 1) [12,16,20-26]. The APACHE II score was evaluated in six studies and showed poor to good discriminating value with areas under the ROC curve between 0.60 and 0.78 [16,20-22,25,26]. Discrimination of the SAPS II model was fair to good with areas under the ROC curve between 0.67 and 0.83 [20-23,25,26]. The MPM II model showed poor discrimination in one study [12], but good discrimination in another [26]. Discrimination of all models differed importantly between studies. All models showed fair to good discrimination in the study by Soares [26] and poor discrimination in the study by Sculier [20]. This may be related to differences in casemix of patients; whereas most patients in the latter trial had metastatic or disseminated haematological disease, most patients in the study by Soares had locoregional cancer or cancer in remission only.

In 1998, Groeger and others developed a model specific for cancer patients [12]. It included physiological data, disease-related variables (allogeneic bone marrow transplantation and recurrent or progressive cancer), and performance status before hospitalization. Tested on an independent set of patients, the model showed good discriminating power with an area under the ROC curve of 0.81. Good discriminating ability was confirmed in two other studies [22,26]. In 2003, Groeger *et al. *developed another specific model with good discriminating performance (AUC = 0.82) that predicts in-hospital mortality in cancer patients at 72 h after ICU admission [13].

### Calibration

As shown in Table 1, most studies showed poor calibration for APACHE II and III, SAPS II and MPM II [12,20,22,26]. The studies that have good H- or Ĉ-statistics (*p*>0.05) are very small. Poor calibration resulted in a uniform underestimation of the mortality risk using the general prognostic models. Hence, the observed mortality was uniformly higher than the predicted mortality (SMR>1). In contrast with the general prognostic models, the specific models for cancer patients showed good calibration with SMR of 1.0 to 1.05 [12,13,21] or poor calibration resulting in a uniform overestimation of mortality risk (SMR 0.75 [26]).

### Identification of subgroups at (very) high risk

It may be particularly important to identify patients at very high risk of dying. Patients with limited life expectancy do not necessarily prefer life-extending treatment over care focused on relieving pain and discomfort. The willingness to receive life-sustaining treatment depends on the burden of treatment, the outcome and the likelihood of the outcome [27]. In patients aged 65 years and older, the willingness to receive cardiopulmonary resuscitation if they suffered a cardiac arrest decreased from 41 to 22% after learning the probability of survival (10 to 17%) [28].

Although none of the selected studies report on accuracy measures, some studies report on the performance of prognostic models at specific cut-off points for predicted mortality. Results are summarized in Table 2. High predicted mortality by the general prognostic models as well as the specific ICU Cancer Mortality Model (ICCM) was associated with very high observed mortality rates. For example, in a study by Staudinger and others, 7% of the studied population had more than 79 APACHE III points, and all of these patients died before hospital discharge [24]. In another study by Sculier and others, 5.4% of patients had a predicted mortality of >70% according to the APACHE II model. In these patients, the actual observed mortality was 86% [20]. Thus, in a limited number of cancer patients, a very high mortality chance after ICU admission may be predicted. It may be speculated that some patients would prefer not to undergo intensive care treatment if their predicted mortality is very high, for example, more than 80 or 90%. Providing prognostic information to patients, their relatives and physicians could help to provide intensive care that is more in accordance to patients' own preferences.

## Discussion

The general prognostic models for ICU patients generally underestimate the risk of dying for cancer patients admitted to ICUs. This is important when interpreting SMRs of different ICUs, since ICUs with relatively more cancer patients will have a higher SMR. However, these models are able to identify subgroups of patients with a very high mortality risk. Thus, they may have a role in giving information about the prognosis to patients and their relatives.

Only a few models exist that were specifically designed for cancer patients and which include data about type and stage of cancer, and functional status of patients. These models showed better discrimination and calibration than the general models. Thus, they may have a role in comparing SMRs of cancer patient populations in the ICU. However, they have been validated in relatively few patients and new larger studies are required to confirm the value of these models. Most studies had a retrospective design and limited number of patients, and the moderate differences among the scoring systems do not allow conclusion of the superiority of one of them. Because of large variations in their design (type of patients, observed mortality (33 to 60%), mix of H- and Ĉ-statistics), it is difficult to perform meaningful comparisons between them. Different casemixes, national or regional patient populations and critical care management can lead to different outcomes.

A limitation of all models is the fact that they do not take into account that better treatments may become available and that prognosis may improve over time. Indeed, it has been shown that survival of patients after haematopoietic stem cell transplantation who received mechanical ventilation, improved from lower than 10% in the period before 1990 to 25 to 50% in the period 1994 to 2000 [16]. Thus, prognostic information should be interpreted cautiously. Nevertheless, patients and physicians need optimal information about the likelihood of a beneficial outcome of intensive care treatment. Prognostic models do at least contribute to this information.

## Conclusion

The general prognostic models for ICU patients generally underestimate the risk of dying for cancer patients admitted to ICUs. Models specific for cancer patients show better calibration and discrimination than the general models. Both general models and specific oncology models may reliably identify subgroups of patients with a very high mortality risk and thus may be useful to inform patients and their relatives about the likelihood of a beneficial outcome.

## Key messages

• 	General prognostic models for ICU patients, such as APACHE II or SAPS II, tend to underestimate the risk of dying for patients with cancer admitted to ICUs.

• 	Prognostic models specifically designed for ICU patients with cancer show better calibration and discrimination than the general models.

• 	Both general models and specific oncology models reliably identify subgroups of patients with a very high risk of dying.

## Abbreviations

APACHE = Acute Physiology and Chronic Health Evaluation; AUC, area under the curve; ICCM = ICU Cancer Mortality Model; ICU, intensive care unit; MPM = Mortality Probability Model; ROC = receiver operating curve; SAPS = Simplified Acute Physiology Score; SMR = standardized mortality rate.

## Competing interests

The authors declare that they do not have competing interests.

## Authors' contributions

SdB and EdJ acquired and interpreted the data. NFdK interpreted the data. All authors contributed in preparing the manuscript. All authors read and approved the final manuscript.

## Figures and Tables

**Table 1:** Overall predictive performance of prognostic models in ICU patients with cancer

					Mortality%		
						
Study	N Solid/metastatic/haematological malignancies	Prognostic score	ROC	Hosmer-Lemeshow goodness-of-fit H or Ĉ test *p *value	Pred	Obs	SMR
Sculier [20]	261	APACHE II	0.60	0.001 (H)	26.5	33	1.25
	solid 77%	SAPS II	0.67	0.001	26.1		1.26
	metastatic 62%						
	haematological 23%						
Groeger [12]	805	MPM II	0.63	<0.001 (Ĉ)	22	41	1.86
	230	ICMM	0.81	0.310	ng		1.02
	solid and haematological						
Groeger [13]	415	ICMM at 72 h	0.82	0.354 (Ĉ)	ng	ng	1.0
	solid and haematological						
Schellongowki [21]	242	APACHE II	0.78	0.058 (Ĉ)	ng	44	1.05
	solid 45%	SAPS II	0.83	0.066	ng		
	haematological 55%	ICMM (2)	0.70	0.115	42		
Berghmans [22]	247	APACHE II	0.65	0.002 (H)	32	34	1.06
	solid 80.5%	SAPS II	0.72	<0.0001	24		1.42
	metastatic 62%	ICMM (2)	0.79	0.060	28		1.21
	haematological 19.5%						
Guiguet [23]	94	SAPS II	0.78	0.750 (H)	ng	60	1
	solid 44.7%						
	haematological 55.3%						
Staudinger [24]	414	APACHE III	0.75	ng	ng	47	
	solid 42%						
	haematological 58%						
Benoit [25]	124	SAPS II	0.77	0.60 (Ĉ)	ng	54	
	haematological 100%	APACHE II	0.71	0.39	ng		
Afessa [16]	112	APACHE III	0.70	0.584 (H)	42	46	1.09
	haematological 100%	APACHE II	ng	ng	44		1.03
Soares [26]	542	SAPS II	0.82	<0.001 (H)	47.9	58.7	1.23
	solid 88.8%	APACHE III	0.81	<0.001	42.6		1.38
	haematological 11.2%	ICMM (2)	0.80	<0.001	78.7		0.75
	excluding scheduled	MPM II 24 h	0.79	<0.001	37.7		1.56
	surgical patients	APACHE II	0.75	<0.001	38.2		1.54
		MPM II 0 h	0.73	<0.001	25.0		2.35

Shown are areas under ROC, *p *value belonging to Hosmer-Lemeshow goodness-of-fit H- or Ĉ- statistics and SMRs for individual mortality prediction models. APACHE, Acute Physiology and Chronic Health Evaluation; ICMM, ICU Cancer Mortality Model; MPM, Mortality Probability Model; ROC, receiver operating curve; SAPS, Simplified Acute Physiology Score; SMR, standardized mortality rate; ng, not given.

**Table 2:** Positive predictive value of prognostic models at specific cut-off values for predicted mortality by severity-of illness models

Study	Prognostic model and cut-off probability of mortality	Observed Mortality, %
Sculier [20]	APACHE II >70%	86
	SAPS II >60%	67
Berghmans [22]	APACHE II >60%	78
	SAPS II >60%	86
	ICMM >40%	67
Staudinger [24]	APACHE III ≥ 79*	100
Kroschinsky [29]	SAPS II ≥ 80*	95
Groeger [13]	ICCM_72 _>70%	83
Groeger [12]	ICCM >80%	91

*Severity of illness scoring points, not predicted mortality. APACHE, Acute Physiology and Chronic Health Evaluation; ICCM, ICU Cancer Mortality Model; ICCM_72_, ICU Cancer Mortality Model at 72 h; SAPS, Simplified Acute Physiology Score.
